# A machine learning model to determine the accuracy of variant calls in capture-based next generation sequencing

**DOI:** 10.1186/s12864-018-4659-0

**Published:** 2018-04-17

**Authors:** Jeroen van den Akker, Gilad Mishne, Anjali D. Zimmer, Alicia Y. Zhou

**Affiliations:** Color Genomics, 831 Mitten Road, Burlingame, CA 94010 USA

**Keywords:** Next generation sequencing, Variant calling, Variant quality, Variant confidence, Secondary confirmation, Orthogonal confirmation, Machine learning, Sanger sequencing

## Abstract

**Background:**

Next generation sequencing (NGS) has become a common technology for clinical genetic tests. The quality of NGS calls varies widely and is influenced by features like reference sequence characteristics, read depth, and mapping accuracy. With recent advances in NGS technology and software tools, the majority of variants called using NGS alone are in fact accurate and reliable. However, a small subset of difficult-to-call variants that still do require orthogonal confirmation exist. For this reason, many clinical laboratories confirm NGS results using orthogonal technologies such as Sanger sequencing. Here, we report the development of a deterministic machine-learning-based model to differentiate between these two types of variant calls: those that do not require confirmation using an orthogonal technology (high confidence), and those that require additional quality testing (low confidence). This approach allows reliable NGS-based calling in a clinical setting by identifying the few important variant calls that require orthogonal confirmation.

**Results:**

We developed and tested the model using a set of 7179 variants identified by a targeted NGS panel and re-tested by Sanger sequencing. The model incorporated several signals of sequence characteristics and call quality to determine if a variant was identified at high or low confidence. The model was tuned to eliminate false positives, defined as variants that were called by NGS but not confirmed by Sanger sequencing. The model achieved very high accuracy: 99.4% (95% confidence interval: +/− 0.03%). It categorized 92.2% (6622/7179) of the variants as high confidence, and 100% of these were confirmed to be present by Sanger sequencing. Among the variants that were categorized as low confidence, defined as NGS calls of low quality that are likely to be artifacts, 92.1% (513/557) were found to be not present by Sanger sequencing.

**Conclusions:**

This work shows that NGS data contains sufficient characteristics for a machine-learning-based model to differentiate low from high confidence variants. Additionally, it reveals the importance of incorporating site-specific features as well as variant call features in such a model.

**Electronic supplementary material:**

The online version of this article (10.1186/s12864-018-4659-0) contains supplementary material, which is available to authorized users.

## Background

Recent advances in next generation sequencing (NGS) have led to a rapid increase in the development and utilization of NGS-based clinical tests. Previously, clinical genetic tests used capillary-based Sanger sequencing, and this technology has been considered the gold-standard for decades. Therefore, as clinical labs transitioned to NGS assays, it became standard to confirm variants identified by NGS with a secondary, orthogonal method such as Sanger sequencing [1, 2]. However, the field does not currently agree on the accuracy and reliability of NGS assays and the necessity of orthogonal confirmation [3], with some studies advocating that orthogonal confirmation is unnecessary and potentially detrimental [4, 5], some advocating that confirmation is always necessary [6, 7], and others suggesting that confirmation is required for some but not all variants [8, 9].

The data generated by a capture enrichment NGS assay can vary greatly across parameters such as read depth, sequencing quality, and mapping accuracy. Subsequently, the confidence at which variants can be identified varies widely. For most variants the evidence supporting a call is very strong, and it is therefore highly unlikely that an orthogonal technology would contradict the NGS call [9]. However, some variants in low quality or difficult to sequence regions are more uncertain and should require confirmation. Developing a robust and reliable method for differentiating between these high and low confidence variant calls is essential to create an NGS assay with the highest possible accuracy for clinical testing. Such a method could pinpoint the variants which require orthogonal confirmation, ensuring that those variants are reported correctly. However, recent work has shown that the use of a single value to estimate the quality of a call cannot reliably eliminate the need for confirmation [8]. Other multi-parameter algorithms, such as Variant Quality Score Recalibration, have been developed to assay the quality of NGS variant calls but are designed for whole exome sequencing and whole genome sequencing-sized data sets, and have not been optimized for smaller targeted panels [10].

Here, we present how a machine learning model can reliably identify variants that require additional quality testing. This model analyzes variants called in a capture enrichment-based NGS assay and differentiates between high confidence variants that do not require further orthogonal confirmation and low confidence variants that do. The model uses machine learning to combine multiple features including reference sequence characteristics and variant call quality signals to reliably identify variant calls of high confidence, which are therefore expected to be confirmed as present using an orthogonal technology such as Sanger sequencing. This model was tuned to eliminate false positives, defined as variants erroneously identified as high quality variants that are found to be not present during orthogonal confirmation, a highly undesirable outcome that could result in falsely reporting an incorrect clinical result. The model was used to analyze a set of 7179 variants identified by NGS and re-tested by Sanger sequencing. It categorized 92.2% of the variants as high confidence, and 100% of these were confirmed to be present by Sanger sequencing. Together these results show that while most NGS calls do not require orthogonal confirmation, there still exists a small subset that do, and a machine learning model can reliably identify those important variants.

## Methods

### NGS variant calling

Here, we analyze a set of 7179 variant calls identified by a targeted NGS assay across 6074 samples. The variants include single nucleotide variants (SNVs) and insertions/deletions (indels) ≤ 25 base pairs identified in a 30-gene panel test for hereditary cancer risk adapted from [11]. The 30 genes in this assay were selected for their association with an elevated risk for breast, ovarian, colorectal, melanoma, pancreatic, prostate, uterine, and stomach cancer. The genes included are listed in (Additional file 1: Table S1). The majority of these genes are assessed for variants within all coding exons (+/− 20 bp flanking each exon); exceptions are noted in the table. Additionally, non-canonical splice regions are included. Target regions were enriched by Agilent SureSelect (v1.7), and sequencing was performed by Illumina NextSeq 500 (paired-end 150 bp, High Output kit). The bioinformatics analysis pipeline aligned reads against GRCh37.p12 with the Burrows-Wheeler Aligner (BWA-MEM) [12] and called variants using the GATK3 HaplotypeCaller module [10, 13, 14]. Coverage requirements were a minimum of 20 unique reads (20X) for each base of the reportable range, and at least 50X for 99% for the reportable range. Median coverage was in the 200-300X range. All variants included in this analysis were classified according to ACMG guidelines [15] as variant of uncertain significance (VUS), likely pathogenic, or pathogenic. The variants were identified in 6074 non-consecutive clinical samples from individuals that underwent genetic testing at Color Genomics (Burlingame, CA, USA). DNA was extracted from blood or saliva samples collected using the Oragene DX 510 saliva collection device (DNA Genotek, Ottawa, ON, Canada). Library preparation was performed using Kapa Biosystems HyperPlus reagents (Wilmington, MA, USA). Orthogonal confirmation of variants identified by NGS was performed by Sanger sequencing. Variants identified as present by NGS but not present by Sanger sequencing were tested by Sanger sequencing on at least two distinct amplicons.

### Model development

For each variant (listed in Additional file 2: Table S2), homopolymer length and GC content were calculated based on the reference sequence. Next, for each carrier of a variant, multiple NGS quality signals were collected. All features are summarized in Table 1, including some features used in published variant callers (e.g. [16]). The features belong to one of two broad categories: those providing information about the genomic position in which the call was made (such as GC content and presence of nearby homopolymers), and those directly reporting the expected quality of the call (such as the quality scores estimated by the caller). A subset of the data (70%) was then used to train a logistic regression model against a binary target as to whether the given variant called by NGS had subsequently been confirmed as present using Sanger sequencing. The outcome is a probabilistic estimation of the likelihood of orthogonal confirmation of the NGS call. The model is deterministic in that for the same input it will always produce the same prediction. Subsequently, 15% of the dataset was used for development, and 15% was used for testing the model.

**Table 1:** Features used in the logistic regression model

Feature	Description	Value range: 5th–95th percentile (median)
DP	NGS read depth at the variant position.	78–433 (222)
AD	Number of reads that support the variant call.	25–393 (110)
AF	Fraction of reads that support the variant call, i.e. AD / DP.	0.13–0.56 (0.49)
GC @ 5, 20, 50	Fraction of GC content in the 5, 20, and 50 bases around the variant position.	0.18–0.73 (0.45) 0.29–0.69 (0.44) 0.30–0.68 (0.42)
MQ	Root Mean Square of the mapping quality of the call.	59.3–60 (60)
GQ	Genotype Quality of the call.	50–99 (99)
WHR	Weighted Homopolymer Rate in a window of 20 bases around the variant position: the sum of squares of the homopolymer lengths, divided by the number of homopolymers.	1.6–4.3 (2.4)
HPL-D	Distance to the longest homopolymer within 20 bases from the call position.	0–15 (5)
HPL-L	Length of the longest homopolymer within 20 bases from the call position.	2–6 (4)
QUAL	Quality score assigned by the GATK HaplotypeCaller to the call.	142–5448 (2564)
QD	QUAL, normalized by DP.	1.6–16.9 (11.3)
FS	Phred-scaled *p*-value using Fisher’s exact test, to detect strand bias.	0–9.2 (1.7)

Names and descriptions of features incorporated into the model. For each feature, the median and range as the 5th–95th percentile of values within the dataset is reported

In this context, a false positive prediction is a variant detected by NGS that was predicted to be high-quality but was not detected by Sanger sequencing. A false negative prediction is an NGS call that was identified as low confidence, but for which the Sanger sequencing identified the variant as present. With the task at hand, false positive predictions are significantly more costly than false negative predictions: while a false negative, which is a truly high confidence call being incorrectly identified as low confidence, introduces some delay to completing the analysis due to potentially unnecessary Sanger sequencing, a false positive, which is a low confidence call being incorrectly identified as high confidence, can lead to an incorrect result reported if Sanger sequencing were not used to test and remove the low confidence NGS variant. We implemented this model in a clinical laboratory setting, and therefore biased the model to prefer the consequence of a false negative (performing more Sanger sequencing than is potentially necessary) to the consequence of a false positive (clinically reporting a variant that does not exist). As such, the model was tuned to eliminate false positive predictions. Further details about the implementation of this machine learning model in a clinical laboratory are included in Additional file 3, including a discussion of concept drift and investigation of discordant cases by trained experts.

## Results

### Model performance

**Fig. 1:**
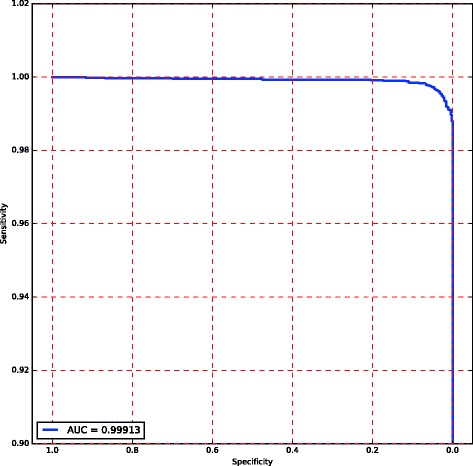
Model Sensitivity. ROC curve plotting the true positive rate (sensitivity) against the false positive rate (specificity). The model threshold was chosen to eliminate false positives (variants erroneously called as high-confidence, but were found to be not present by Sanger sequencing). This resulted in a calculated area under the curve (AUC) of 0.99913

We developed and trained a machine learning model to differentiate between high confidence NGS calls that do not require orthogonal confirmation and low confidence NGS calls that do require confirmation. Due to the strong correlation between the features used for the model and the quality of an NGS call, the model achieves very high accuracy: 99.4% (95% confidence interval: +/− 0.03%). The sensitivity and specificity of the model plotted in Fig. 1 show an area under the curve (AUC) of 0.999. As with any model that outputs a probabilistic score, a threshold can be set on the model’s prediction to trade-off between true positive prediction rate and false positive prediction rate: a high threshold increases sensitivity at the cost of specificity, and vice versa. Due to the high cost of false positive predictions, we set a strict threshold to achieve a 100% true positive prediction rate: any NGS call that the model predicts as high confidence is indeed confirmed, but some of the NGS calls that the model indicates as low confidence (requiring orthogonal confirmation) are confirmed present too. The data presented in Fig. 1 represents the model performance on the 15% of the data held-out for model testing.

The results of a 10-fold cross-validation of the model on all data are shown in Table 2. We performed orthogonal confirmation by Sanger sequencing for all variants in the data set, and compared the outcome of Sanger sequencing to the model’s prediction. Of all 7179 variants we analyzed, the model predicted 6622 (92.2%) to be of high confidence, and 100% of those were confirmed to be true positives. The model predicted 557 variants to be of low confidence, and 92.1% of those were true negatives (513/557). Only 44 of the NGS variants called at low confidence were confirmed by Sanger sequencing (0.6% of all 7179 variants). Previous methodologies have been reported to encounter difficulties with indels [8]. In contrast, our model was able to effectively differentiate high and low confidence indels: 100% (1138 of 1138) of high confidence indels were true positive predictions, and 1.9% (4 of 212) of low confidence indels were false negative predictions (Additional file 2). Lastly, only 7.8% (557 of all 7179 variants) were classified as low confidence, indicating that most variants can be called reliably by NGS alone.

**Table 2:** Model performance

	Present	Not present
High confidence variant	6622 / 6622 (100.0%) *True positive prediction*	0 / 6622 (0%) *False positive prediction*
Low confidence variant	44 / 557 (7.9%) *False negative prediction*	513 / 557 (92.1%) *True negative prediction*

Results of a 10-fold cross-validation of the model on all 7179 variants. Variants called in the NGS pipeline were tested by Sanger sequencing. Those that were confirmed by Sanger sequencing are reported here as “Present”, and those that did not confirm are reported here as “Not Present”. All the variants were evaluated by the machine learning model and categorized as a “High confidence variant” or a “Low confidence variant”

### Key model parameters

Previous studies have attempted to differentiate between true and false NGS calls, employing various protocols and parameters to make this distinction [8, 9]. Our model incorporated the parameters listed in Table 1 to differentiate between high and low confidence variants. We investigated the influence of some key parameters to elucidate how our multi-featured model can accurately assign a confidence level to a variant.

**Fig. 2:**
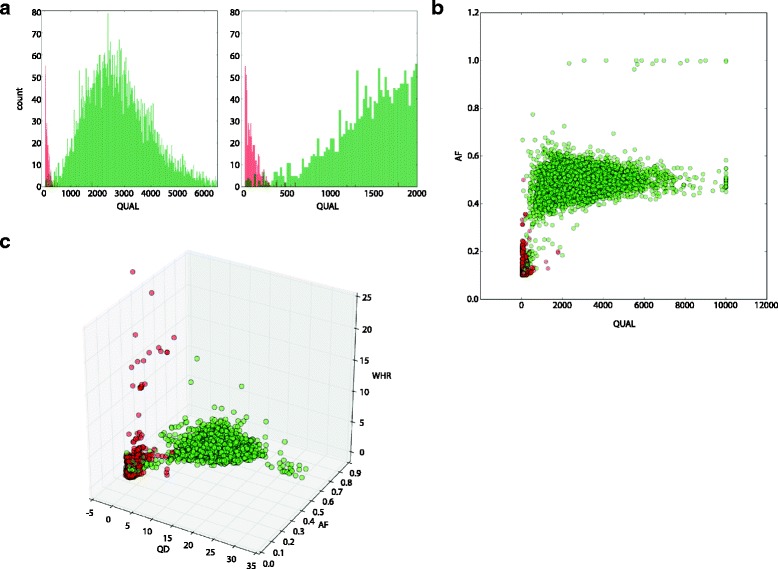
Key model parameters. Predictive power of call-dependent and site-dependent signals. All instances of the data set are shown; instances marked in green are NGS calls that were confirmed with Sanger, and instances marked in red are cases that did not confirm. **a** Call quality (QUAL), a single metric of call quality as measured by GATK HaplotypeCaller. Left panel: all variants. Right panel: zoom-in of variants which did not confirm, showing that some not confirmed variants have relatively high QUAL scores. **b** The values of the two strongest features associated with the call: allele frequency (AF) and QUAL. **c** The values of QUAL normalized by read depth (QD), AF, and weighted homopolymer rate (WHR)

Some studies have used a single indicator of call quality, such a Phred quality score [8], to determine high quality variants. We found that using the single parameter of call quality (QUAL, as assigned by the GATK HaplotypeCaller) is not sufficient to segregate true positives from true negatives with confidence. Figure 2a shows the distribution of call quality, with the variants confirmed present by Sanger sequencing indicated in green and the variants that did not confirm in red. As shown in the zoomed-in second panel of Fig. 2a, there were several variants in our data set with a relatively high QUAL score (> 1500) that did not confirm (colored in red). In order to eliminate false positives, all variants with a score below 2000 would require confirmation, necessitating orthogonal confirmation of 32% of variants (2338/7179).

In our analysis, the most influential parameter was allele frequency (AF), or the fraction of reads that support the call, calculated by dividing the number of reads that support the call by the total read depth at the position (Fig. 2b). While AF does a relatively good job of segregating high and low confidence alleles, the introduction of additional parameters greatly improves the performance of the model, as can be seen in Fig. 2b, where the two most influential parameters, call quality and allele frequency, are combined.

Normalizing QUAL by read depth (QD = QUAL/DP) and inclusion of the homopolymer rate surrounding the variant (WHR) results in further segregation of confirmed and not confirmed variants (Fig. 2c). In its entirety, the model incorporates all parameters listed in Table 1. Material improvements were achieved with the relatively minor cost of incorporating these additional features, which are standardly collected by our bioinformatics pipeline.

### Model performance on challenging variants

**Fig. 3:**
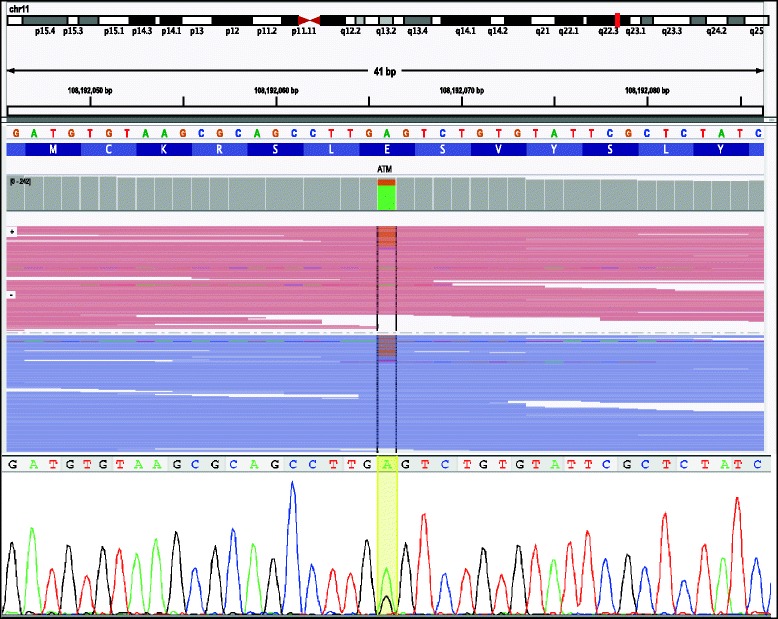
High confidence, low allele fraction variant. Example of a true positive prediction of a low allele fraction variant, *ATM* (c.6491A > G). Top: NGS reads, visualized in IGV [18, 19] showing the variant was detected in reads of both directions. Bottom: Sanger sequencing chromatogram, showing a detected allele fraction of ~ 25%

As shown in Fig. 2b, allele fraction is a key feature that can be used to distinguish between high and low confidence variants. However, by incorporating many features, the model still has the ability to classify low fraction variants as high confidence. For example, Fig. 3 shows a variant with an allele fraction of ~ 25% in *ATM* (c.6491A > G) that was confirmed to be at a low fraction by Sanger sequencing (bottom panel). This variant was given a relatively low call quality score by GATK (QUAL = 683.77). However, the high coverage in the region (DP = 175) and good quality of the site (balanced GC content, no homopolymers, no strand bias) resulted in the model correctly predicting this as a high confidence variant. This example highlights that including site-specific features is important for good model performance, especially for challenging variants.

**Fig. 4:**
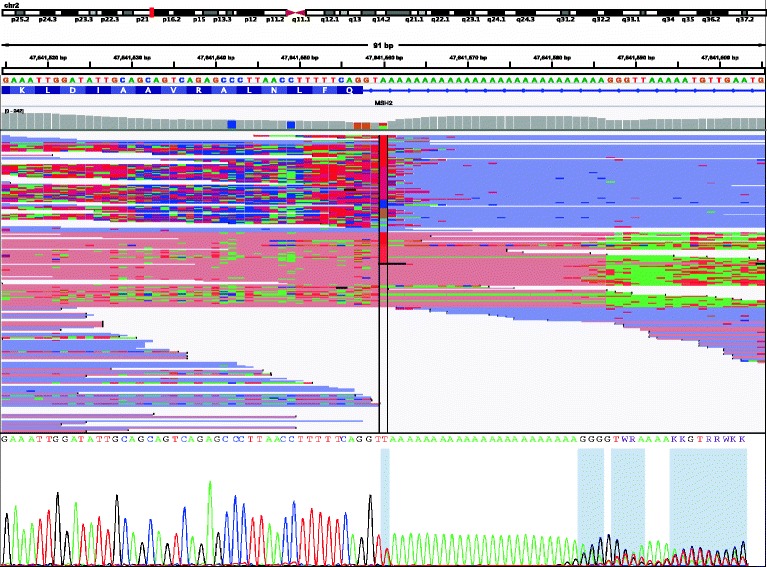
False negative prediction. *MSH2* (c.942 + 3A > T), an example of a false negative prediction. Top: NGS reads, visualized in IGV. Note the presence of the long homopolymer results in errors in sequencing and alignment. Bottom: Sanger sequencing chromatogram, showing the presence of the variant

In contrast, Fig. 4 shows an example of a variant that the model determined to be of low confidence but which was actually confirmed by Sanger sequencing. This variant in *MSH2* (c.942 + 3A > T) was challenging to detect due to the presence of a long homopolymer and highlights the necessity of orthogonal confirmation to ensure accurate calls for low confidence, clinically-actionable variants.

Overall in the dataset, the rate of low confidence variants that were confirmed by Sanger sequencing was low (7.9%), representing 0.6% of all variants. However, we wanted to understand the reason for the low confidence estimated by the model in these cases. Variants were therefore annotated for repetitive elements and genomic duplications (> 1000 bp and > 90% identity) using the UCSC genome browser tracks for Repeatmasker and Segmental Duplications respectively. In addition, variants attributed to the presence of processed pseudogenes were flagged. We then examined the subset of variants that the model predicts as low-quality (requiring confirmation) that were actually confirmed using Sanger sequencing (Table 3). A majority of these variants were low fraction, called in substantially less than 30% of the NGS reads and visible as lower peaks in Sanger chromatograms. Other common features of these variants were those typically difficult for NGS sequencing, such as having high or low GC content or being in close proximity to long homopolymers.

**Table 3:** Features of low confidence variants

Low Confidence	Present (*n* = 44)		Not Present (*n* = 513)	
	AF < 30% (37)	AF ≥ 30% (7)	AF < 30% (505)	AF ≥ 30% (8)
Low coverage (20–30 reads)	0	2	15	2
Low GC content (GC20 < 0.25)	2	2	23	2
Low GC content (GC50 < 0.25)	1	0	5	0
High GC content (GC20 > 0.75)	1	0	206	1
High GC content (GC50 > 0.75)	0	0	35	1
Homopolymer (> = 10 within 20 bp)	0	3	15	7
Segmental duplication	16	4	67	0
Processed pseudogene	0	0	3	0
Repeatmasker	0	2	6	0
Other	17	2	246	0

Common features of variants classified by the model as low confidence. “Present” indicates that the variant was confirmed present by Sanger sequencing, and “Not Present” indicates that the variant was not present by Sanger sequencing. The number of low confidence variants with AF (allele frequency) less than and greater than or equal to 30% are reported in parentheses. Note that some of the categories are not mutually exclusive

## Discussion

The rise of NGS in genetic testing has lowered cost and increased accessibility of testing. Several recent studies have investigated the accuracy of variant calling in NGS pipelines, and reconsidered the necessity of confirming variants with an orthogonal technology. Here, we presented a machine-learning-based model that can differentiate between high confidence variants that do not, and low confidence variants that do, require orthogonal confirmation.

We developed and tested our model on a set of 7179 variants identified in a capture-based assay that had been classified as pathogenic, likely pathogenic, or variants of uncertain significance. This set included low quality variants that are difficult to call in NGS data such as low fraction variants. Nonetheless, our model was able to accurately segregate high and low confidence variants for not only SNVs, but also for the more challenging indels [8]. Importantly, this model was able to achieve high accuracy using data from a small 30-gene panel, in contrast to previous methods which require much larger datasets [10]. Indeed, in our setting, we found that performance of the model started converging after about 100 true negatives and 2000 true positives.

Low coverage variants, variants in regions of high homology, and low fraction variants are usually classified by the model as low confidence variants that require confirmation. Here, we highlighted a variant in *MSH2* (c.942 + 3A > T) that the model identified as low confidence, but which Sanger sequencing confirmed to be present (Fig. 4). This variant has been previously reported and discussed in the literature as a difficult variant for NGS assays. A recent report [5] suggested that dedicated algorithms can reliably call such challenging variants in NGS data. However, the results from this study and others [6] support the necessity of Sanger confirmation in such difficult-to-sequence regions, especially when relying on variant callers that have not been optimized for such regions.

Though the model described here can be employed as a useful tool for identifying low confidence variants, it does have limitations that would hinder its use to evaluate all variant types. Importantly, the model must be trained on the types of variants that are to be analyzed. If others were to adapt the techniques presented here, we strongly recommend that they optimize the model on a training set that closely matches the variants they plan to analyze. In this work the model training set was limited to small variants (≤25 bp) that are typically confirmed by Sanger orthogonal confirmations, and thus was unable to assess variants that might be detected by other methodologies such as alternative sequencing technologies, MLPA, and array CGH. Additionally, while the training set did include homozygous variants and low fraction variants, these variants are technically challenging, relatively rare, and clinically interesting. Therefore in our application of the model in a clinical setting, we choose a conservative approach for these variants and always orthogonally confirm homozygous variants, low fraction variants (AF 0.1–0.3), and variants in regions where a processed pseudogene was detected in the same sample. Lastly, we developed the model with germline variants only, and its applicability to somatic variants has not been tested.

The model presented here captures and evaluates the potentially problematic features associated with a variant call and determines the likelihood that the variant is truly present. Not surprisingly, low allele fraction, GC content, and segmental duplications account for most of these low confidence variants, as these genomic regions are known to be difficult for NGS and lead to inaccurate variant calling. This only reinforces the importance of confirming variants in difficult regions, despite recommendations by other groups that confirmation of NGS results is not necessary [4, 5]. Continued confirmation will build additional data that can be used to evaluate and improve NGS variant calling for low allele fraction variants and somatic variants. This study and others [8, 9, 17] add to a growing body of evidence that the majority of variants calls in NGS data alone are accurate, and the practice of orthogonal confirmation for all variants may not continue to be standard in the field. As the limitations of NGS technology are better studied and understood, appropriate safeguards can be enacted where necessary.

## Conclusions

While the cost of NGS has dropped dramatically over the past decade, including orthogonal confirmation of all calls may be adding unnecessarily to the cost of genetic testing. Most NGS calls are of high quality, and our model classified 92% of the variants in this study as high confidence variants. While just using one indicator of call quality was not sufficient to make this distinction, the combination of many measures of call quality allowed a machine learning model to make a clear differentiation between high and low confidence variants. The majority of low confidence variants had features known to be problematic for capture-based NGS assays: low allele fraction, segmental duplications, and aberrant GC content. The model performed with high accuracy on both SNVs and indels. Using this model to robustly differentiate high and low confidence variants could reduce the burden of necessary orthogonal confirmations, while identifying the few calls that do need to be confirmed to maintain the high quality required of a clinical genetic test.

## Additional files

Additional file 1: **Table S1.** Genes in panel. Genes tested in the 30-gene NGS panel. Variants in genes indicated with an asterisk (*) were not evaluated by the machine learning model because only large copy number variants were evaluated in these genes. (XLSX 27 kb)

Additional file 2: **Table S2.** Variant Dataset. Characteristics of variants used for model development and testing. (XLSX 844 kb)

Additional file 3: **Supplemental Methods.** Using the model in a clinical laboratory setting. (DOCX 103 kb)

## Abbreviations

indel: Insertion/deletion
NGS: Next generation sequencing
SNV: Single nucleotide variant
VUS: Variant of uncertain significance
