# Supplementary material for: A machine learning model to determine the accuracy of variant calls in capture-based next generation sequencing
Source: BMC Genomics. 2018 Apr 17;19:263. doi: 10.1186/s12864-018-4659-0 (PMC5904977; doi:10.1186/s12864-018-4659-0)
Supplement: Supplementary file 3 — Supplemental Methods. Using the model in a clinical laboratory setting. (DOCX 103 kb) [file 12864_2018_4659_MOESM3_ESM.docx]

Supplemental Methods

Using the model in a clinical laboratory setting

Applying a machine-learned model in a clinical lab setting requires extra rigor to ensure consistent high-quality model performance. In particular, we discuss two issues and their solutions:

Concept Drift

In supervised machine learning, it is common for the data used to build a statistical model to change over time. For example, improvements to the assay, changes in the types of samples processed, and upgrades of the software used to process them may result in different value distributions of the features used for the model. A model trained once on a particular set of data points may be less accurate when applied to data obtained later, when these distributions changed. Several approaches exist in the machine-learning world to address Concept Drift, most focusing on re-training or updating the model regularly [1].

Furthermore, as additional samples are processed by the lab through an NGS assay and more variants are orthogonally confirmed with Sanger sequencing, more data is available to tune the model. The performance of any statistical model is directly tied to the amount of data used to create the model, as well as its consistency and quality, and it is common to see improvements in model accuracy as additional data is used to build it — even if value distributions are stable.

The model described in this paper can easily be periodically updated with new data. Its accuracy is continuously measured using cross-validation, monitored using dashboards, and in cases of underperformance, an alert triggers further investigation.

Discordant cases

As defined earlier, false positive predictions are NGS variants predicted by the model to be high-quality, but not detected by Sanger sequencing; false negative predictions are NGS variants predicted to be low-quality (and require Sanger confirmation), but for which the Sanger method actually agreed with the NGS call. With high-accuracy models such as the one described here, it can be appealing to rely on the model to completely replace expert judgment. However, monitoring and surfacing both types of false predictions offers opportunities for identifying model limitations and improving its performance, as well as improving the underlying assay itself. An automated system continuously monitoring discordant predictions has been implemented for the model described in this paper, so that when Sanger sequencing of an NGS-called variant is complete, its result is immediately compared to the prediction, triggering an alert for false predictions. Alerts are investigated by domain specialists to uncover model limitations.

Reference

1. Gama J, Žliobaitė I, Bifet A, Pechenizkiy M, Bouchachia A. A Survey on Concept Drift Adaptation. ACM Comput. Surv. New York, NY, USA: ACM; 2014;46:44:1–44:37.
